# WGS- versus ORF5-Based Typing of PRRSV: A Belgian Case Study

**DOI:** 10.3390/v13122419

**Published:** 2021-12-02

**Authors:** Frank Vandenbussche, Elisabeth Mathijs, Marylène Tignon, Tamara Vandersmissen, Ann Brigitte Cay

**Affiliations:** 1 Unit Exotic Viruses and Particular Diseases, Scientific Directorate of Infectious Diseases in Animals, Sciensano, 1180 Brussels, Belgium; 2 Unit Enzootic, Vector-Borne and Bee Diseases, Scientific Directorate of Infectious Diseases in Animals, Sciensano, 1180 Brussels, Belgium; Elisabeth.Mathijs@sciensano.be (E.M.); Marylene.Tignon@sciensano.be (M.T.); AnnBrigitte.Cay@sciensano.be (A.B.C.); 3 Animal Health Care Flanders (DGZ), 2500 Lier, Belgium; Tamara.Vandersmissen@dgz.be

**Keywords:** porcine reproductive and respiratory syndrome virus, whole-genome sequencing, genotyping, recombination

## Abstract

Porcine reproductive and respiratory syndrome virus (PRRSV) is the causative agent of one of the most widespread and economically devastating diseases in the swine industry. Typing circulating PRRSV strains by means of sequencing is crucial for developing adequate control strategies. Most genetic studies only target the highly variable open reading frame (ORF) 5, for which an extensive database is available. In this study, we performed whole-genome sequencing (WGS) on a collection of 124 PRRSV-1 positive serum samples that were collected over a 5-year period (2015–2019) in Belgium. Our results show that (nearly) complete PRRSV genomes can be obtained directly from serum samples with a high success rate. Analysis of the coding regions confirmed the exceptionally high genetic diversity, even among Belgian PRRSV-1 strains. To gain more insight into the added value of WGS, we performed phylogenetic cluster analyses on separate ORF datasets as well as on a single, concatenated dataset (CDS) containing all ORFs. A comparison between the CDS and ORF clustering schemes revealed numerous discrepancies. To explain these differences, we performed a large-scale recombination analysis, which allowed us to identify a large number of potential recombination events that were scattered across the genome. As PRRSV does not contain typical recombination hot-spots, typing PRRSV strains based on a single ORF is not recommended. Although the typing accuracy can be improved by including multiple regions, our results show that the full genetic diversity among PRRSV strains can only be captured by analysing (nearly) complete genomes. Finally, we also identified several vaccine-derived recombinant strains, which once more raises the question of the safety of these vaccines.

## 1. Introduction

Porcine reproductive and respiratory syndrome virus (PRRSV) is the causative agent of one of the most widespread and economically devastating diseases in the swine industry [1]. Although the disease emerged nearly simultaneously in Europe and North America, virus strains from both continents are surprisingly divergent, sharing only about 60% nucleotide identity [2]. As a consequence, both genotypes were recently reclassified within the *Betaarterivirus* genus of the Arteriviridae family as two separate species: *Betaarterivirus suid 1* (formerly known as PRRSV-1 or PRRSV-EU) and *Betaarterivirus suid 2* (formerly known as PRRSV-2 or PRRSV-NA) [3,4]. For convenience, we will use the commonly accepted traditional names, PRRSV-1 and PRRSV-2, throughout the rest of the text.

Similar to other members of the *Betaarterivirus* genus, PRRSV is an enveloped, positive-sense, single-stranded RNA virus with a genome of approximately 15 kb in length encoding at least 10 open reading frames (ORFs 1a, 1b, 2a, 2b, 3, 4, 5a, 5, 6, and 7) [1,5]. The replicase machinery is encoded by the overlapping ORFs 1a/1b, which comprise roughly 77% of the genome. Both ORFs are translated into large polyproteins (pp1a and pp1ab), which are proteolytically processed into 14 non-structural proteins. The structural proteins are encoded by the eight remaining ORFs (ORFs 2a-7) located downstream of ORF1b. Similar to most viruses in the Nidovirales order, the structural proteins are expressed through a set of 5′–3′ co-terminal subgenomic RNAs (sgRNAs) using a discontinuous transcription strategy [6,7]. The synthesis of these sgRNAs is mediated by transcription regulatory sequences (TRS), a series of conserved nucleotide sequences that are located in the 5′-untranslated region of the genome (leader TRS) and upstream of all genes encoding the structural proteins (body TRSs). The interaction between the viral RNA-dependent RNA polymerase (RdRP) and the body TRS elements results in either read-through or dissociation of the minus-strand sgRNA template from the RNA genome. Reassociation of the minus-strand sgRNA-RdRP complex with the leader TRS then generates the typical 5′-3′ co-terminal, minus-strand sgRNA molecules. The resulting minus-strand RNA molecules subsequently serve as a template for the production of positive-strand genomes and subgenomic messenger RNAs (sgmRNAs) [5].

Control of PRRSV through vaccination has proven to be particularly challenging as none of the current modified live-attenuated vaccines (MLV) offer broad cross-protection against heterologous strains [8,9,10]. Moreover, several reports have revealed safety issues including shedding and persistent MLV infections, reversion to virulence, and recombination between MLV and field strains [9]. Effective prevention and control strategies require a more holistic approach combining proper biosecurity measures, herd management, systematic monitoring, and vaccination.

Typing circulating PRRSV strains through sequencing is a key factor in developing adequate control strategies. Sequencing not only allows discriminating vaccine-like from wild-type strains, but it can also help to establish whether an outbreak is due to the introduction of a new strain or recirculation of a previous strain. Genetic studies of PRRSV strains have been based largely on the highly variable ORF5, which encodes the major envelope glycoprotein (GP5). Over the years, several typing methods have been proposed to classify PRRSV-1 strains. Using a neighbour-joining tree inference method, Stadejek et al. divided PRRSV-1 strains into four subtypes based upon the observed ORF5 tree topology [11]. Shi et al. applied an alternative approach using Bayesian tree inference combined with average pairwise genetic distance thresholds to define 12 clades within subtype 1 [12]. More recently, Lambert et al. described an automated classification system that is based on maximum likelihood tree inference and a maximum pairwise genetic distance threshold [13]. Besides ORF5, ORF7 size polymorphism has been proposed as an alternative PRRSV-1 subtype marker [14]. Although ORF5 remains the most common target for PRRSV sequencing and phylogenetic analyses, ORF5-based typing does not allow for capturing the full genetic variation of PRRSV strains. The ORF5 sequence accounts for merely 5% of the entire PRRSV genome and thus can only provide a very limited snapshot of the entire genetic variation [1,15]. Typing based on complete genomes or multiple protein-coding regions could provide a more comprehensive picture of the genetic relatedness of PRRSV strains [1,15,16].

Unfortunately, sequencing viral genomes directly from clinical samples remains challenging due to the high abundance of non-viral nucleic acids. To overcome this challenge, most of the earlier protocols used a long-range PCR strategy to generate a limited number of overlapping amplicons covering the entire genome [17]. More recently, the emergence of high-throughput sequencing (HTS) has made it possible to reconstruct PRRSV genomes by sequencing the entire RNA content of a sample without a pre-amplification step. To increase the proportion of viral reads, samples are either subjected to rRNA depletion [18] or sequenced to a high read depth [19]. In this study, we performed cDNA sequencing on a collection of 124 PRRSV-1 positive serum samples that were collected over a 5-year period (2015–2019) in Belgium. The aim of the study was twofold: (1) to assess the added value of whole-genome sequencing (WGS) as a PRRSV-typing tool and (2) to characterize the contemporary Belgian PRRSV population.

## 2. Materials and Methods

### 2.1. Samples

All serum samples were collected by the official herd veterinarians as part of a national monitoring program (PRRS-Programme: Health Monitor Piglets) [20]. Samples were taken from 3–12-week-old piglets over a 5-year period (2015–2019) and originated from 77 pig farms that were all located in the Flemish-speaking part of Belgium. An initial screening on pooled serum samples (pool size of 5) was performed by DGZ using the VetMAX PRRSV EU & NA kit (Thermo Fisher Scientific, Waltham, MA, USA). Individual samples of pools with a Cp value below 30.00 were retested to identify the most positive sample(s). More details of the samples can be found in the Supplementary Materials (Table S1).

### 2.2. RNA Extraction and DNase Treatment

Nucleic acids were extracted from serum samples using a NucleoSpin RNA Virus Mini kit (Macherey-Nagel, Düren, Germany) according to the manufacturer’s instructions but without the addition of carrier RNA to the RAV1 lysis buffer. Contaminating DNA was removed by treating all the extracts with Baseline-ZERO DNase (Lucigen, Middleton, WI, USA). The remaining RNA was purified and concentrated into a final volume of 15 µL using an RNA Clean & Concentrator-5 kit (Zymo Research, Irvine, CA, USA).

### 2.3. cDNA Synthesis

First strand cDNA was synthesised from 10 µL purified/concentrated RNA using an anchored oligo(dT) primer (5′-(T)47AATTDCGG-3′), a PRRSV-specific internal primer (5′- GCGRCCACAGCGG-3′), and SuperScript IV Reverse Transcriptase (Thermo Fisher Scientific) according to the manufacturer’s instructions. Second-strand cDNA synthesis was performed using the NEBNext Ultra II nondirectional RNA second-strand synthesis module kit (New England Biolabs, Ipswich, MA, USA). The resulting cDNA was purified and concentrated into a final volume of 13 µL using a DNA Clean & Concentrator-5 kit (Zymo Research, Irvine, CA, USA).

### 2.4. High-Throughput Sequencing

Sequencing libraries were prepared using the Nextera XT DNA Library Preparation Kit (Illumina, San Diego, CA, USA) according to the manufacturer’s instructions with slight modifications. To account for differences in cDNA yield, the number of PCR cycles was adjusted on a sample-to-sample basis ranging between 12 and 15 cycles. The fragment length distributions of the libraries were verified on a Bioanalyzer System (Agilent Technologies, Santa Clara, CA, USA). Libraries were quantified using a KAPA Library Quantification Kit (Roche, Basel, Switzerland) and pooled equimolarly. Sequencing pools were analysed on a MiSeq System (Illumina, San Diego, CA, USA) using the MiSeq Reagent Kit version 3 (Illumina) with 2 × 300-bp paired-end sequencing, yielding approximately 1 million read pairs per sample.

Adapter sequences and low-quality bases were removed with Trimmomatic v0.38 using the MAXINFO adaptive quality trimming criterion [21]. Prior to de novo assembly, datasets were enriched for PRRSV using mirabait (MIRA v5rc1) with a target dataset containing all available (nearly) complete PRRSV genomes from GenBank [22]. Enriched datasets were subsampled to 12,500, 25,000, 50,000, or 100,000 reads per sample using Seqtk v1.3 (https://github.com/lh3/seqtk, accessed on 14 December 2018) and assembled with IVA v1.0.8 [23], MIRA v5rc1 [22], and SPAdes v3.9.0 [24] using default parameters. The resulting contigs were combined into a single consensus sequence, and nucleotide variants were called using the GATK best practices pipeline v4.1.3.0 [25,26]. Finally, all genomes were annotated with VADR [27] using a local PRRSV model and deposited in GenBank under accession numbers MZ417390 to MZ417500.

### 2.5. Datasets

All available (nearly) complete PRRSV-1 genomes were retrieved from GenBank on 25 November 2020. Duplicate sequences were removed using the UCLUST algorithm (USEARCH version 11.0.667) with an identity threshold of 1.00 [28]. The final unaligned dataset (*n* = 245) was obtained by merging the remaining GenBank sequences (*n* = 134) with all Belgian sequences (*n* = 111) from the current study.

Two types of aligned datasets were used throughout the study: a WGS dataset and a series of ORF datasets. To detect potential recombination events, the entire dataset was first reduced with Treemmer v0.3 using the CDS phylogenetic tree as input tree and a relative tree length of 0.95 [29]. All available PRRSV-1 MLV genomes (i.e., Porcilis PRRS vaccine: DD093450, UNISTRAIN PRRS vaccine: GU067771, Ingelvac PRRSFLEX EU vaccine: KT988004, Suvaxyn PRRS MLV: MK876228) were included in the dataset to facilitate the detection of vaccine-derived recombinants. The pruned dataset (*n* = 145) was aligned with MAFFT v7.475 using the E-INS-i algorithm. As the 5′- and 3′-untranslated regions were missing from most of the genomes, both terminal untranslated regions were removed from the aligned dataset to obtain the final WGS dataset.

All phylogenetic and clustering analyses were performed on ORF datasets. Open reading frames ORF1a, ORF1b, ORF2a, ORF3, ORF4, ORF5, ORF6, and ORF7 were extracted from the unaligned dataset and stored in separate ORF datasets. Each ORF dataset (*n* = 245) was aligned using the AlignTranslation and StaggerAlignment functions from the DECIPHER package [30]. Finally, the resulting alignments were concatenated into a single CDS dataset (*n* = 245) using the FastaCon python script (https://github.com/Pas-Kapli/FastaCon, accessed on 12 June 2020).

### 2.6. Distribution of Insertions and Deletions

Gap positions and sizes were extracted from the aligned, non-staggered ORF datasets using the DNAbin2indel function from the ape package [31]. The resulting data were condensed into a gap pattern, which describes all gaps of a given PRRSV strain in a single string. Each non-empty gap pattern contains the starting point of the gap (number following the prefix ‘P’) as well as the size of the gap (number following the colon character). Multiple gaps are ordered according to their starting point and separated by a slash character. For instance, gap pattern ‘P160:6/P187:3′ represents a 6 bp deletion at position 160 and a 3 bp deletion at position 187. To facilitate comparison of gap patterns on a genomic scale, a numeric identifier was assigned to each unique gap pattern. For each PRRSV strain, the gap pattern identifiers of the different ORFs were concatenated into a single string using a slash character as a separator. The resulting gap code thus describes all insertion and deletion events (InDels) of a PRRSV strain. For instance, gap code ‘33/7/1/5/3/1/1/2’ represents a PRRSV strain containing gap pattern 33 for ORF1a, gap pattern 7 for ORF1b, etc.

### 2.7. Phylogenetic Analysis

Prior to phylogenetic analysis, each dataset was partitioned by codon position and subjected to the MaxSymTest analysis as implemented in IQ-TREE v2.1.0 [32]. Partitions that failed the analysis were removed from the dataset. Optimal partitioning schemes and nucleotide substitution models were selected using ModelFinder v2.1.0 [33,34]. Presence of phylogenetic signal was evaluated by likelihood mapping analysis [35] using the optimal partitioning schemes and nucleotide substitution models. Trees were reconstructed using the maximum likelihood (ML) method as implemented in IQ-TREE v2.1.0 [36]. For each dataset, 50 independent runs were performed using the best partitioning schemes and nucleotide substitution models as determined before. Branch supports were assessed using UFBoot2 [37] as well as SH-aLRT [38] with 10,000 replicates. The tree with the highest ML-score was selected and annotated in the R environment using the package ggtree [39].

### 2.8. Cluster Analysis

Phylogenetic trees were subjected to cluster analysis according to the methodology described by Prosperi et al. [40]. In brief, trees were analysed using a depth-first algorithm, which starts at the root node and explores each subtree as far as possible along the branches before backtracking. A subtree was considered to be eligible as a cluster only if it met the following criteria: (i) it contained at least 2 PRRSV strains, (ii) it had a node reliability (UFBoot value) above a bootstrap threshold of 90%, and (iii) the mean of its pairwise patristic distance distribution was below a t-percentile threshold of the whole-tree distance distribution. Whenever these criteria were fulfilled in a node, the search at that node was stopped, and all child nodes were ignored. These steps were then repeated on the remaining node siblings until the entire tree had been traversed. For each dataset, the t-percentile threshold was optimized over the 1st–10th percentile range of the whole-tree distance distribution with a step size of 0.25 using the maximum number of clustered PRRSV strains as the optimization criterion. Cluster analysis was implemented in the R environment using the packages ape [31], dplyr [41], geiger [42], igraph [43], treeio [44], and xlsx [45].

To facilitate comparisons between datasets, CDS clusters were renumbered according to decreasing size, while ORF clusters were renumbered to maximize overlap with the renumbered CDS clusters. Clustering schemes of the ORF datasets were compared with the CDS clustering scheme, which served as reference, as it contained the genetic information of all ORFs. To visually assess the (dis)similarity between schemes, the renumbered CDS and ORF clusters of all PRRSV strains were plotted against each other using ggplot2 [46]. Due to the renumbering, highly similar clustering schemes will yield a strong linear relationship with only a limited number of outliers. The CDS and ORF clustering schemes were also compared statistically using the adjusted mutual information (AMI) [47] and the BCubed F-score (Fb^3^) [48]. Both similarity metrics were calculated in the R environment using the packages aricode [49] and DPBBM [50], respectively.

### 2.9. Recombination Analysis

Potential recombination events in the WGS dataset were identified using RDP5.05 as described in the user manual [51]. Most parameters were left at their default settings, except for the sequence type (set to linear) and the highest acceptable *p*-value (set to 0.05). Sequences were considered to be recombinant when the same recombination signal was identified by at least 4 methods.

To analyse genome-wide recombination patterns, a recombination region count matrix was constructed in RDP5.05. This matrix indicates how often genomic sub-regions are separated from one another by recombination and thus highlights the exchangeability of sequence tracts within the PRRSV genome. Highly exchangeable regions are represented by warm colours, whereas less exchangeable regions are represented by cool colours.

The distribution of recombination breakpoints was visualized by plotting all breakpoint locations on a density map. A permutation test was used to identify statistically significant hot- or cold-spots in the breakpoint distribution. To investigate a potential association between body TRS elements and recombination hot-spots, TRS-like core sequences were identified in the dataset with Vmatch v2.3.1 (http://www.vmatch.de/, accessed on 30 March 2020) using the hexanucleotide motif [A/U][A/U/C][A/G][A/C]CC as query sequence. The positions of the 6 major body TRS motifs and the TRS-like motifs were plotted on top of the breakpoint distribution plot using ggplot2 [46].

## 3. Results

### 3.1. Whole Genome Sequencing Analysis

One hundred and twenty-four Belgian PRRSV-1 positive serum samples collected over a 5-year period (2015–2019) were analysed by high-throughput sequencing. Using a cDNA sequencing approach, we obtained nearly complete genomes, including all 10 ORFs, from 111 serum samples corresponding to a 90% success rate (sequencing reports available upon request). The proportion of reads derived from the PRRSV target varied substantially across the samples, ranging from 0.15% to 75.60%. In general, the number of PRRSV reads tended to increase as the PRRSV viral load increased. In nine samples, the viral load was too low (qRT-PCR Cp values > 30) to generate sufficient PRRSV reads for genome assembly. As expected, *Sus scrofa* was the most abundant species in the majority of the samples. Eleven samples were heavily contaminated with reads of bacterial origin (> 50% of total reads), which greatly reduced the number of PRRSV reads. As a consequence, we were unable to reconstruct a complete PRRSV genome in three of these samples. Although mixed infections were rare, we found multiple PRRSV-1 strains in three samples and a combination of PRRSV-1 and PRRSV-2 strains in seven samples. All of the PRRSV-2 strains were highly similar to PRRSV-2 vaccine strains, showing ≥ 99% nucleotide identity at the genome level. Due to the high genetic relatedness, we failed to recover the genomes of individual strains from mixed PRRSV-1 samples. By contrast, the strains from mixed PRRSV-1/PRRSV-2 samples could be easily distinguished from one another (data not shown).

Two strains originating from different farms (BEL-WVL-2018-S04, and BEL-WVL-2018-S07) displayed a genetic distance lower than 1% compared to the VP-046-BIS strain (UNISTRAIN PRRS vaccine). Most remaining strains differed substantially from the available strains in GenBank with a mean genetic distance at the genome level of 19.21% (range: 6.71–29.72%). In fact, only strain BEL-WVL-2018-S21 exhibited a genetic distance below 10% and was found to be most similar to the DV strain (Porcilis PRRS vaccine). The genetic diversity was also high among the Belgian strains themselves with a mean genetic distance at the genome level of 18.31% (range: 0.04–22.55%). Several nearly identical genomes (genetic distance < 1%) were found among the Belgian strains. Most of these were collected from the same farm or from nearby farms.

Marked differences in pairwise genetic distance were observed between the various ORFs of the Belgian, non-Belgian, and complete datasets. As the datasets were non-random with some countries/provinces being overrepresented, both the mean and maximum pairwise genetic distances were calculated for each dataset. ORF1a, ORF3, ORF4, and ORF5 were the most divergent, but the ranking differed depending on the measure that was used, while ORF6 and ORF7 were the most conserved (Table S2).

### 3.2. Insertions and Deletions

To facilitate the analysis of size polymorphisms, unique gap patterns were identified for each PRRSV strain and ORF dataset (File S1). Although size polymorphisms were observed in nearly all ORFs, most InDels were found in ORF1ab, ORF3, and ORF4 (Table 1). Fifty-six InDels were identified in the Belgian ORF1ab dataset, with 50 internal InDels being located in the NSP2 protein. A total of 23 different NSP2 size variants were observed ranging from 1003 aa to 1078 aa. The remaining six InDels, one internal and five terminal, were located in the NSP12 protein. All Belgian PRRSV strains contained multiple InDels which resulted in 38 different ORF1ab gap patterns. Considerable size polymorphism was also observed in the Belgian ORF3 dataset, which contained 9 internal and 12 terminal InDels. No fewer than 22 distinct ORF3 gap patterns and 17 GP3 size variants were identified (225–269 aa). As all InDels were positioned toward the end of ORF3, several of them directly impacted the size of the overlapping ORF4. Analysis of the Belgian ORF4 dataset revealed 11 internal InDels and 12 gap patterns. Although size polymorphism in ORF4 was markedly lower than in ORF1ab and ORF3, seven GP4 size variants were observed (172-183 aa). An internal InDel (3 bp) in ORF6 was observed in two Belgian PRRSV strains that originated from the same farm and sampling date. Finally, no InDels were found in the Belgian ORF5 and ORF7 datasets.

### 3.3. Recombination Analysis

Using a series of recombination detection methods, we identified 125 unique recombination events in the WGS dataset (File S2). More than half of the sequences in the dataset appeared to be recombinant. Recombination events were detected in 41% of the non-Belgian sequences and 76% of the Belgian sequences. In 15 events, at least one of the parental strains was a vaccine (similarity ≥ 99.5%) or a vaccine-like (97.5 ≤ similarity < 99.5%) strain. All four vaccine strains present in the dataset were involved in one or more recombination events. Four recombinant genomes, including one Belgian sample, contained traces of multiple vaccine strains (Table 2).

**Figure 1:**
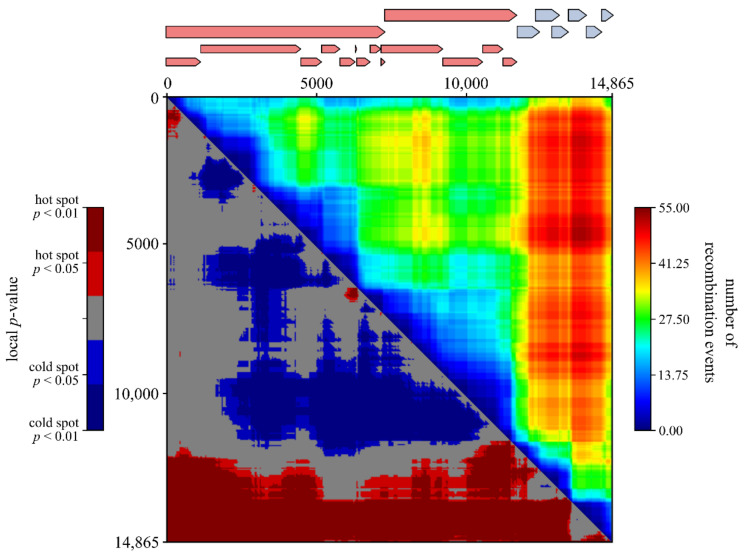
Recombination region count matrix (upper right) and recombination region hot/cold spot matrix (lower left) of the WGS dataset. Unique recombination events were mapped onto the matrices based on their estimated breakpoint positions. The colours in the upper right matrix are a function of the number of times that pairs of nucleotides (plotted on the x and y axes) are separated by observable recombination events. Highly exchangeable regions are depicted by warm colours, whereas less exchangeable regions are depicted by cool colours. The blue and red colour in the lower left matrix represent pairs of sites that appear to be the least and most separable by recombination. The genome organization is represented on top of the graph. Non-structural and structural proteins are shown in red and blue, respectively.

**Figure 2:**
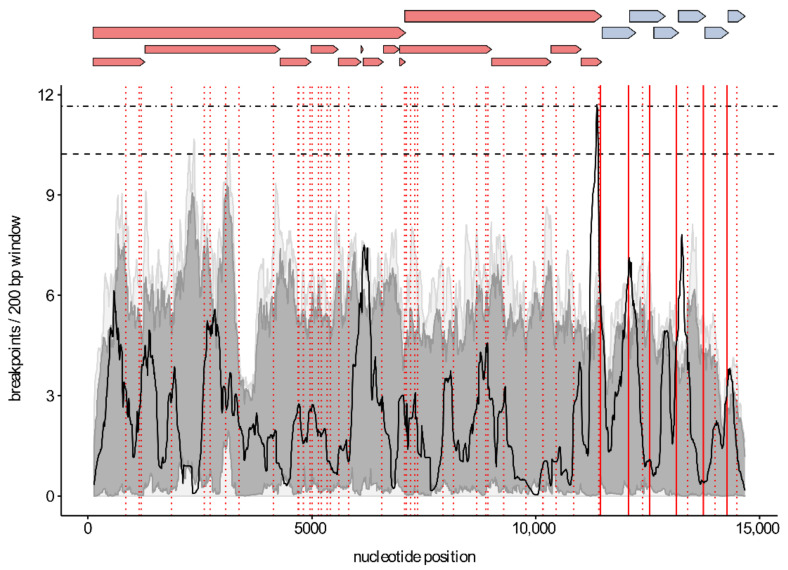
Distribution of recombination breakpoints within the WGS dataset. A 200 bp window was moved along the alignment 1 bp at a time, and the number of breakpoints detected within the window region was counted and plotted (solid lines). The horizontal upper and lower dashed lines indicate the 99% and 95% confidence thresholds for globally significant breakpoint clusters. Light and dark grey areas indicate the 99% and 95% local breakpoint clustering thresholds. The positions of body TRS- and TRS-like elements are depicted as vertical lines. The red solid lines represent the body TRS elements in the alignment. The positions of TRS-like elements that are present in at least 33% of the sequences are shown as red dotted lines. The genome organization is represented on top of the graph. Non-structural and structural proteins are shown in red and blue, respectively.

To study whether certain genomic regions are exchanged more often than others, we mapped the unique recombination events onto region count matrices. As shown in Figure 1, the genomic region containing the structural proteins (ORF2-ORF7) is exchanged most frequently during recombination. We also visualized the distribution of the recombination breakpoints by plotting all breakpoint locations on density maps (Figure 2). A globally significant recombination hot-spot (global *p*-value < 0.01) is present toward the end of NSP12. In addition, locally significant recombination hot-spots (local *p*-value < 0.01) were found in the NSP1, NSP7α, GP2/GP3, GP3/GP4, GP4/GP5, and M/N regions. To further investigate whether breakpoints tend to cluster within ORFs, we performed a binary variable test in RDP5 which will test for associations between breakpoint locations and genome regions. The results of these association tests showed that recombination breakpoints cluster more frequently within the beginning and ending 10% of ORFs (*p*-value < 0.001). Finally, by plotting the positions of body TRS elements on top of the breakpoint distribution plot, we found indirect evidence to link body TRS elements with recombination, as four of the six major body TRS elements were located near breakpoint hot-spots (solid red lines in Figure 2). Similar body TRS-like elements were found across the entire genome. Although the association is not that strong, most of these elements also tended to cluster with regions of increased recombination activity (dotted red lines in Figure 2).

### 3.4. Phylogenetic Analysis of Separate versus Concatenated ORFs

To gain more insight in the added value of WGS, we analysed all ORFs both in separate datasets and as part of a single, concatenated dataset comprising all ORFs. Substitution saturation analysis indicated that datasets ORF1a, ORF1b, ORF4, ORF6, ORF7, and CDS experienced little saturation at any of the codon positions (I_ss_ < I_ss.c_; *p*-value < 0.001). Analysis of the ORF2a, ORF3, and ORF5 datasets revealed weak signs of saturation at the third codon position. Although all I_ss_ values were smaller than the I_ss.c_ values, the difference was no longer significant when the true tree topology was extremely asymmetric and the number of OTUs was greater than 16. As such an asymmetric topology is not realistic for PRRSV sequences, we decided not to exclude the third codon position from the datasets.

Likelihood mapping analyses revealed marked differences in the phylogenetic content of datasets (Table 3). As expected, the longest datasets contained the highest phylogenetic signal (CDS > ORF1a > ORF1b). Although the remaining datasets were much smaller, most of them still contained a sufficient amount of tree-likeness with more than 75% of all quartets being fully resolved in the ORF5, ORF3, and ORF6 datasets.

### 3.5. Phylogenetic Cluster Analysis

Maximum likelihood-based phylogenetic analyses were performed on each dataset, and the resulting phylogenetic trees were subjected to cluster analysis according to the methodology described by Prosperi et al. [40].

**Figure 3:**
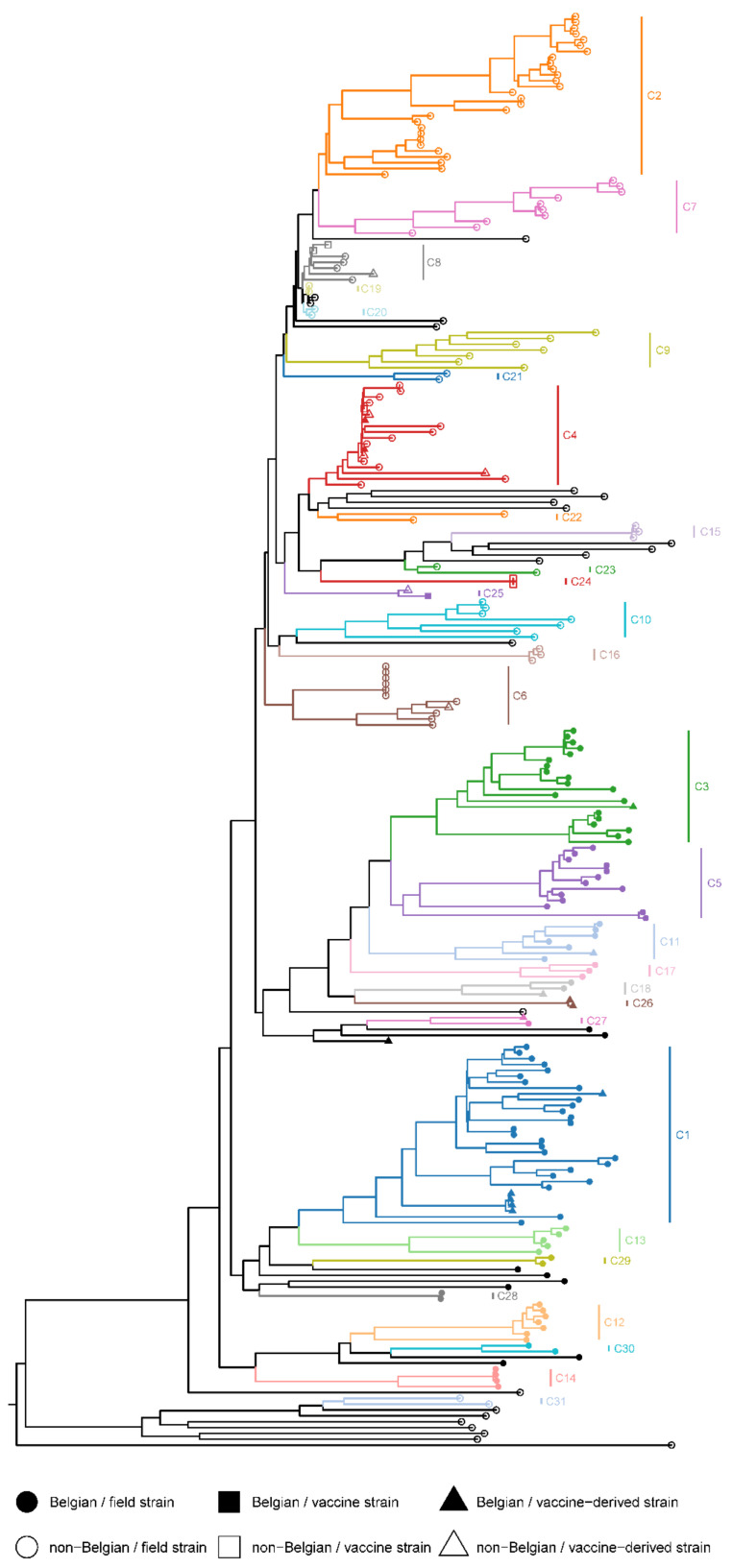
Maximum likelihood tree of the CDS dataset. The phylogenetic tree was reconstructed using the maximum likelihood (ML) method as implemented in IQ-TREE v2.1.0 [36]. Optimal partitioning schemes and nucleotide substitution models were selected using ModelFinder v2.1.0 [33,34]. Fifty independent runs were performed using the best partitioning schemes and nucleotide substitution models. Branch supports were assessed using UFBoot2 [37] as well as SH-aLRT [38] with 10,000 replicates. The tree with the highest ML-score was mid-point rooted and annotated in the R environment using the packages phytools [52] and ggtree [39], respectively. The tree was coloured according to the CDS clustering scheme (C1–C31).

Thirty-two PRRSV-1 strains, including nine Belgian strains, could not be clustered in the CDS dataset because of low bootstrap support values or too high phylogenetic distances (Figure 3). The remaining 213 PRRSV-1 strains were assigned to 31 CDS clusters (Table S3). The Belgian PRRSV-1 strains were divided over 16 CDS clusters, most of which only contained Belgian strains. The two clusters that contained non-Belgian strains were both vaccine clusters. The two Belgian strains from CDS cluster 4, BEL-WVL-2018-S04 and BEL-WVL-2018-S07, were nearly identical to the VP-046-BIS strain (UNISTRAIN PRRS vaccine), and the Belgian strain from CDS cluster 25 is, in fact, the 96V198 strain (Suvaxyn PRRS MLV).

**Figure 4:**
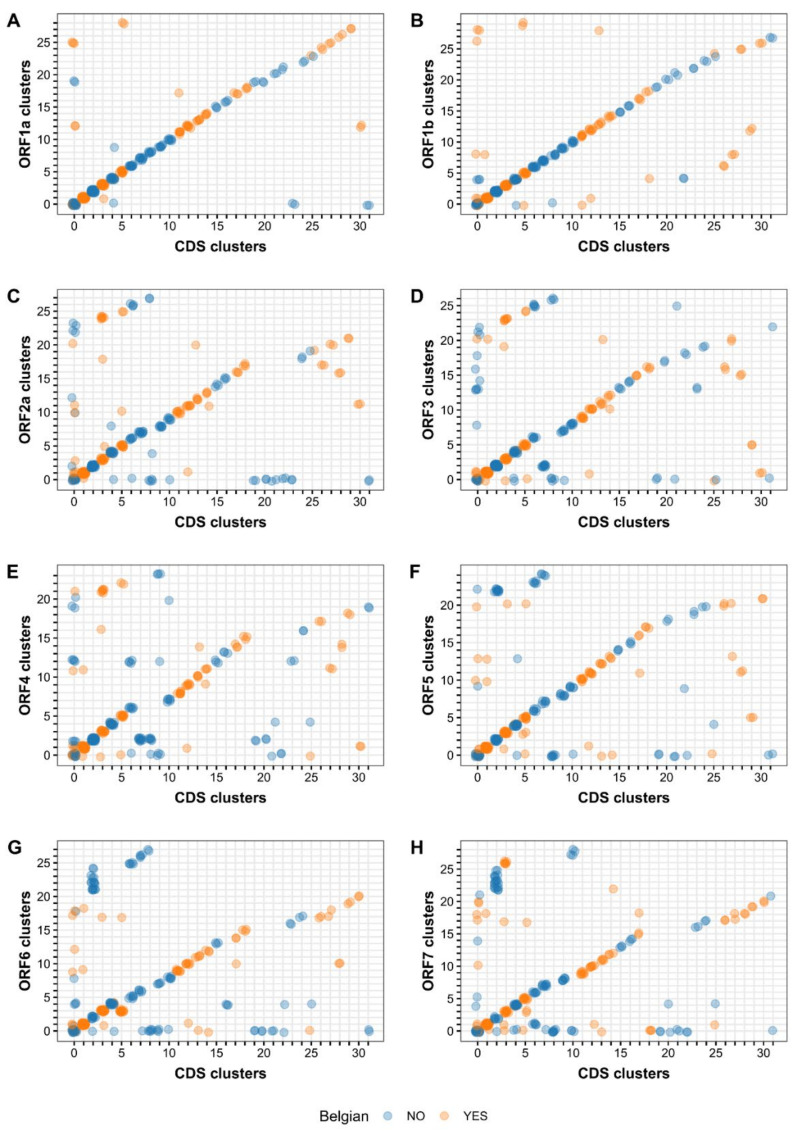
Comparison of the renumbered clustering schemes of the CDS and ORF datasets. Maximum likelihood trees were generated for the CDS and ORF datasets using IQ-TREE v 2.1.0 [36]. The resulting trees were subjected to cluster analysis according to the methodology described by Prosperi et al. [40]. Clustering schemes of the ORF datasets were compared with the CDS clustering scheme, which served as reference. The renumbered CDS and ORF clusters of all PRRSV-1 strains were plotted against each other using the R package ggplot2 [46]. (**A**) Comparison between the ORF1a and CDS clustering schemes; (**B**) Comparison between the ORF1b and CDS clustering schemes; (**C**) Comparison between the ORF2a and CDS clustering schemes; (**D**) Comparison between the ORF3 and CDS clustering schemes; (**E**) Comparison between the ORF4 and CDS clustering schemes; (**F**) Comparison between the ORF5 and CDS clustering schemes; (**G**) Comparison between the ORF6 and CDS clustering schemes; (**H**) Comparison between the ORF7 and CDS clustering schemes.

To evaluate the clustering schemes of the different datasets, all ORF clustering schemes were compared with the CDS clustering scheme which served as the reference scheme. The renumbered CDS and ORF clusters of all PRRSV-1 strains were plotted against each other to visualize differences between clustering schemes (Figure 4). Despite the differences in genetic data that were used for phylogenetic analysis, a linear relationship was observed between the CDS clustering scheme and the different ORF clustering schemes. The ORF1a and ORF1b clustering schemes were most similar to the CDS clustering scheme, showing a clear overlap between the schemes (i.e., linear relationship in the scatter plot) and only a limited number of discrepancies (i.e., outliers in the scatter plot). The ORF4, ORF6, and ORF7 clustering schemes differed most from the CDS clustering scheme with a large number of PRRSV-1 strains being assigned to different clusters in both schemes. The remaining clustering schemes (ORF2a, ORF3, and ORF5) showed intermediate results. The similarity between the CDS and ORF clustering schemes was also assessed using the AMI and Fb^3^ measures, which confirmed the results from the visual analysis. The highest AMI and Fb^3^ values were obtained for ORF1a and ORF1b (Table 4).

## 4. Discussion

Genotyping circulating PRRSV strains by means of sequencing is a key factor in developing effective prevention and control strategies. In this study, we performed cDNA sequencing on a collection of 124 PRRSV-1 positive serum samples that were collected over a 5-year period (2015–2019) in Belgium (sequencing reports available upon request). Although no pre-amplification or rRNA depletion was performed, complete coding regions were obtained for 111 samples using an average sequencing depth of only 1 million paired-end reads per sample. The extent of genome coverage was determined mainly by the PRRSV cDNA load of the sample. In general, whole genome sequences could be obtained for samples with qPCR Cp values ≤ 30. This cut-off is markedly higher than the one reported by Zhang et al., who were unable to reconstruct whole genomes from serum samples with qRT-PCR Cp values ≥ 24.3 [18]. Out of the 13 samples that failed analysis, three were found to be heavily contaminated with bacteria and contained insufficient PRRSV reads to reconstruct the entire genome. Seven samples with qPCR or qRT-PCR Cp values > 31 most likely failed because of insufficient PRRSV particles being present. The remaining three samples were infected by more than one PRRSV-1 strain, which hampered genome reconstruction. Although these samples contained sufficient PRRSV reads, separate genomes could not be assembled accurately because the strains were too closely related. However, it should be noted that all of these mixed samples were readily detected when the raw reads were mapped against the consensus genome. Seven samples were found to be co-infected with both a PRRSV-1 field strain and a PRRSV-2 vaccine-like strain. In contrast to the mixed PRRSV-1 samples, both strains could be easily distinguished from one another, and two separate genomes were obtained for all samples with sufficiently high viral loads (data available upon request). Similar results were described by Zhang et al. [18], who were unable to distinguish a PRRSV-2 field strain from a PRRSV-2 vaccine strain sharing 92.4% nucleotide identity at genome level. These results are not surprising as both protocols use a short-read sequencing platform that is not very well suited to link variants separated by more than the platform’s read length. In theory, long-read sequencing platforms, such as nanopore sequencing by Oxford Nanopore Technologies, should be able to solve this problem, as they can easily produce reads exceeding the size of an entire PRRSV genome. Recently, Tan et al. [53] reported a protocol based on direct RNA sequencing (DRS) using an Oxford Nanopore MinION sequencer. In contrast to other sequencing protocols, DRS analyses RNA molecules directly in their native format as they move through the nanopores. Although Tan et al. were able to distinguish two distantly related PRRSV strains (82.4% nucleotide identity at genome level), analysing more closely related PRRSV strains will be difficult due to the low raw read accuracy of nanopore sequencing. Moreover, the requirement of huge amounts of high-quality poly-adenylated RNA (~500 ng) essentially restricts the use of DRS to virus isolates.

Analysis of the coding regions of all genomes confirmed the exceptionally high genetic diversity among PRRSV-1 strains. Separate analyses were performed on the Belgian (excluding vaccine-like strains) and non-Belgian (subtype I and subtypes I-III) PRRSV-1 strains (Table S2). The diversity within each dataset was measured using the maximum pairwise genetic distance as suggested by Forsberg et al. [54]. Although this measure only describes the genetic extremes of the datasets, it is expected to be less sensitive to sampling bias. Comparison of the Belgian and non-Belgian subtype I PRRSV-1 strains showed that the genetic diversity is nearly identical in both datasets. As reported earlier, the variation within the genome was unequally distributed, and a number of highly variable regions were identified in ORF1a and ORF3–ORF5 [55,56,57].

The high genetic diversity is also apparent when looking at the large number of gap codes that describe the InDel patterns on a genomic scale. As reported earlier, InDels were not distributed randomly across the genome and were located mainly in ORF1a (NSP2), ORF3, and ORF4 [56]. We identified no fewer than 126 different gap codes in the entire dataset. Once again, the diversity in the Belgian dataset (57 gap codes) was nearly as high as in the non-Belgian dataset (70 gap codes). With the exception of one gap code, all Belgian gap codes were unique to the Belgian dataset. The single gap code (75/7/1/33/16/1/1/2) that was shared was found in 6 Belgian strains and 22 non-Belgian strains, including the VP-046-BIS strain (UNISTRAIN PRRS vaccine). As mentioned earlier, two of the Belgian strains were nearly identical to the VP-046-BIS strain (UNISTRAIN PRRS vaccine). However, the remaining four strains are not derived from this vaccine strain. We did not find any signs of genetic exchange with the VP-046-BIS strain (UNISTRAIN PRRS vaccine) in our recombination analysis. Moreover, none of the four strains clustered together with the vaccine strain in our phylogenetic analysis. Our results clearly show that gap patterns or gap codes should not be used as a subtyping tool. The fact that PRRSV strains share the same InDel pattern does not necessarily mean that they are genetically related.

Phylogenetic analysis of the concatenated dataset (CDS) revealed that the non-vaccine-like Belgian strains clustered into three phylogenetic distinct clades that could be further divided into 14 subclades (Table S3). These results suggest that multiple PRRSV-1 incursions occurred in Belgium at some point in time. Previous studies already indicated that more than one PRRSV-1 strain circulated in Europe in the 1990s [57,58,59]. The strict separation between Belgian and non-Belgian clusters should be interpreted with caution due to the limited number of PRRSV genomes that are currently available. BLAST analysis of the separate ORFs revealed strong similarities between Belgian and Dutch PRRSV strains (sequencing reports available upon request). Unfortunately, the sequence data of these Dutch strains was limited to the ORF2a–ORF7 region of the genome [60]. Additional WGS data are needed to better understand the evolution and geographical diversity of European PRRSV strains. Besides these non-vaccine-like strains, we also found a small number of vaccine-like strains (both PRRSV-1 and PRRSV-2). As all samples were collected from piglets, these strains are most likely derived directly from the vaccines that were used. Unfortunately, no information was available regarding the vaccination schedules of the animals. Circulation of PRRSV vaccine strains has been described previously [61,62,63]. Moreover, in a recent study, Eclercy et al. showed that vaccinated pigs not only develop viremia for 3–4 weeks but can also transmit the vaccine strain to naïve animals [64].

To assess the added value of whole-genome sequencing, we repeated the same phylogenetic/clustering analysis for each ORF separately (Table S3). As reported previously, we observed marked differences in tree topology and clustering [55,56,60,65]. A comparison of the CDS versus ORF clustering schemes showed that ORF1a and ORF1b described the overall phylogenetic relationship among PRRSV-1 strains better than the other ORFs (Figure 4). These results are not surprising given that the same datasets were found to contain the highest phylogenetic signal in the likelihood mapping analysis. Interestingly, the ORF2a clustering scheme scored slightly better than the ORF5 and ORF3 clustering schemes, even though the phylogenetic signal of the ORF2a dataset was markedly lower than the ORF5 and ORF3 datasets. Despite the good agreement between the CDS and ORF1a/ORF1b clustering schemes, numerous discrepancies in clustering were observed as can be seen easily on the corresponding CDS-ORF scatterplots or in the cluster codes. Due to the renumbering of the clusters, strains with similar CDS/ORF clustering are expected to form a straight line in the plot. The degree of disagreement between both clustering schemes can thus be assessed by looking at the number of points that are not clustered around this line. These aberrant points can be divided into two categories: (i) points that fail to cluster in one of the datasets but not in the other and (ii) points that cluster differently in both datasets. Examination of the unclustered Belgian PRRSV-1 strains (*n* = 9) from the CDS dataset indicated that most of them (*n* = 6) belong to a subtree that could not be completely resolved (node reliability constraint). The remaining strains (*n* = 3) were not clustered because they were too diverse from the other strains (within-cluster diversity constraint). The inability to resolve a subtree can be explained in several ways. Sequences can be so diverse that the phylogenetic signal becomes obscured by the background noise. Conversely, sequences can be too similar and simply contain an insufficient signal to obtain a reliable phylogeny. Finally, the presence of recombinant sequences can distort both the tree topology and branch lengths.

Using a battery of recombination detection methods, we indeed identified a large number of potential recombination events (PREs, File S2). Approximately 57% of all sequences in the WGS dataset displayed signs of recombination. It is not surprising that the recombination frequency is markedly higher in the Belgian (76%) than the non-Belgian (41%) PRRSV-1 sequences. All of the PRRSV strains from the current study were sampled from a relatively small geographic region and over a short period of time, which greatly increases the chance of detecting PREs. Nevertheless, identification of recombinant/parents is not straightforward due to the high genetic diversity among PRRSV-1 strains and the limited number of available PRRSV-1 genomes. As far as we know, this is the first large-scale recombination analysis of PRRSV-1 strains at the genome level. Previous studies generally focused on a single PRRSV-1 strain [66,67,68,69] and/or were based on much smaller datasets [55,70]. A similar, large-scale analysis was recently described for PRRSV-2 strains from China and the United States of America [19]. Although this study cannot be compared directly with ours due to differences in sampling strategy and recombination detection criteria, it is interesting to note that Yu et al. reported an inter-lineage recombination frequency of 72% in Chinese PRRSV-2 sequences during the 2014–2018 period, which is remarkably close to the recombination frequency we found in the Belgian PRRSV-1 sequences. It remains unclear to what extent our results can be generalized to other PRRSV-1 populations. Regular whole-genome sequencing of PRRSV-1 strains is needed to better understand the role of recombination in PRRSV evolution. Nevertheless, our results clearly show that recombination among PRRSV-1 strains occurs frequently in the field.

As described previously for both PRRSV-1 and PRRSV-2, recombination breakpoints were distributed throughout the entire genome (Figure 2). We found a single global recombination hot-spot (*p*-value < 0.01) toward the end of NSP12 and several local hot-spots (*p*-value < 0.01) in NSP1, NSP7α, GP2/GP3, GP3/GP4, GP4/GP5, and M/N. The observed recombination breakpoint pattern is not in line with earlier PRRSV recombination studies [19,55]. However, as already mentioned above, all of these studies differ in their design and methodology, which makes it difficult to directly compare the results. Moreover, as described by Yu et al., PRRSV recombination patterns are not necessarily conserved and can change substantially over both space and time. For instance, recombination hot-spots were generally not shared between their 1991–2013 and 2014–2018 datasets [19]. As PRRSV does not contain typical recombination hot-spots as seen in other viruses, typing PRRSV strains based on a single ORF is not advisable. A better approach would be to use at least two non-adjacent ORFs showing high genetic diversity (e.g., the nsp2 region of ORF1a and ORF5), as was already suggested by Martin-Valls et al. [55]. Ideally, whole-genome sequences should be used for PRRSV typing, as only this approach allows for fully capturing the genetic diversity among PRRSV strains.

Interestingly, the global hot-spot at the end of NSP12 is located near the junction between the non-structural and structural regions. Similar recombination hot-spots, separating non-structural from structural regions, have been described previously in other virus families including the Picornaviridae [71,72,73,74,75] and Caliciviridae [76,77]. It is now generally accepted that genomes of these virus families function as interchangeable modules rather than as strict genomes. As a consequence, their non-structural and structural genes evolve semi-independently, which explains the inconsistencies found between phylogenetic analyses targeting different regions of the genome. Even though the recombination pattern in our PRRSV-1 dataset is not as clear-cut, the region count matrix (Figure 1) clearly shows that genomic exchanges occur most frequently in the ORF2–ORF7 region. However, in contrast to Picornaviruses and Caliciviruses, the region encoding the structural proteins is usually not exchanged entirely. Recombination breakpoints were found across the entire ORF2–ORF7 region. Interestingly, we observed a tendency for recombination breakpoints to occur more frequently within the beginning and ending 10% of genes rather than in the middle 90% (*p*-value < 0.001). This observation is not rare and has already been described for other viruses including several ssDNA viruses [78] and human immunodeficiency virus [79]. Although numerous recombination events have been described for many virus species, little is known about the underlying processes that determine genome-wide recombination breakpoint patterns. According to the most widely accepted model of RNA recombination, the RdRP switches from one RNA molecule (the donor template) to another (the acceptor template) during RNA synthesis, thereby generating chimeric RNA molecules with mixed ancestry [80,81]. It has been hypothesized that the distribution of breakpoints found in nature can be explained by (i) mechanistic processes that determine the locations of recombination breakpoints across the genome (e.g., extent of local sequence identity between the RNA templates, RNA secondary structure) and/or (ii) a purifying selection that acts against dysfunctional recombinants (e.g., avoids disruption of protein folding, maintains important intragenome interactions) [80,81,82]. As the body TRS elements of PRRSV are involved in template switching during discontinuous transcription, it seems reasonable to assume that the same elements might play a role in recombination as well. We therefore plotted the positions of the major body TRS elements on top of the breakpoint distribution plot. As shown in Figure 2, four of the six major body TRS elements were located near breakpoint hot-spots. In addition to the major body TRS elements, we also identified TRS-like elements across the entire genome and calculated the degree of conservation within the alignment. As expected, most of these elements were much less conserved than the major body TRS elements. Nevertheless, 31 out of 49 TRS-like elements were present in at least 50% of the PRRSV sequences, and four elements were even found in 90% of all PRRSV sequences. Although there is no perfect association, the positions of these TRS-like elements tend to cluster with regions of increased recombination activity. Taken together, our results are consistent with the hypothesis that RNA recombination in Nidoviruses is driven by similar factors and/or signals as discontinuous transcription [6,83,84]. Although the association between breakpoint patterns and TRS elements is certainly intriguing, our study does not provide direct evidence to link body TRS elements with recombination. It is important to realize that most sequences in our dataset are derived from field strains, which are the result of combined mechanistic and viability constraints. In vitro experiments using direct RNA or cDNA sequencing on cells co-infected with two PRRSV strains are needed to investigate the exact role played by TRS elements.

Recombination analyses of the Belgian PRRSV-1 strains revealed multiple mosaic strains that are most likely derived from vaccine or vaccine-like strains. Moreover, repeated sampling in two herds showed that the recombinant strains were circulating, as very similar strains were detected on multiple occasions. As the breakpoints were scattered across the genome, most of these strains can only be detected when using a WGS-based typing approach. Comparison of the cluster codes of the vaccine and vaccine-derived strains clearly shows the added value of WGS. Out of the 12 vaccine-derived recombinants, five strains could be easily identified based on their cluster code, as it overlapped partially with a vaccine strain. The remaining seven recombinants were more difficult to spot in the cluster code. This is not surprising given that we used an ORF-based clustering scheme. Due to the size of ORF1a and ORF1b, most recombination events in these regions are not expected to give the same clustering as the vaccine strains. Clustering schemes based on proteins could partially resolve this.

Although only a single vaccine was involved in most strains, the sample BEL-WVL-2018-S21 contained traces of both the DV strain (Porcilis PRRS vaccine) and the 94881 strain (Ingelvac PRRSFLEX EU, ReproCyc PRRS EU, and ImpranFLEX vaccines). Similar multi-vaccine recombinants have been reported previously in France [67] and Denmark [66]. Interestingly, we discovered one additional multi-vaccine recombinant in our dataset. Isolate HUN60077/16 (MK167464) was originally described as a vaccine-derived recombinant sharing ORF1b and ORF3-ORF7 with the VP-046-BIS strain (UNISTRAIN PRRS vaccine) [69]. However, recombination analysis in RDP5 revealed that the last 750 bp of ORF1a are most likely derived from the DV strain (Porcilis PRRS vaccine). Although we were unable to identify the major parent of ORF1a, we found strong evidence of an additional recombination event with the VP-046-BIS strain (UNISTRAIN PRRS vaccine) at the beginning of ORF1a. Overall, our results confirm that the currently used live-attenuated PRRSV vaccines are all capable of recombining with field strains. Additional research is needed to better understand the potential risk of these vaccine-derived strains and the impact they might have on the diversification and dynamics of the PRRSV population.

It is not clear whether any of the Belgian vaccine-like strains caused disease in the infected herds. Previous studies have shown that vaccine-derived recombinants can result in a more rapid spread and/or increased pathogenicity of the recombinant strains compared to their parental strains [64,85,86,87]. Ideally, recombination between vaccine and field strains should be avoided altogether. As TRS elements play such a crucial role in discontinuous transcription, one obvious approach would be to rewire the transcription regulatory network of live-attenuated vaccines. This would introduce genetic traps into the vaccine genome that are triggered by recombination events with field strains [88]. This strategy was recently described by Graham et al. as a means to increase the safety and stability of live-attenuated coronavirus vaccines [89]. As the authors mentioned, the same strategy should be applicable to other members of the Nidovirales order as well.

## 5. Conclusions

In this study, we have shown that (nearly) complete PRRSV genomes can be obtained directly from serum samples with a high success rate. As expected, the extent of genome coverage was determined mainly by the PRRSV load of the sample. However, the presence of contaminating bacteria or closely related PRRSV strains can hamper genome reconstruction. Analysis of the coding regions confirmed the exceptionally high genetic diversity, even among Belgian PRRSV-1 strains. Phylogenetic analysis of the concatenated dataset showed that the non-vaccine-like Belgian strains clustered into three phylogenetic distinct clades that could be further divided into 14 subclades. A large-scale recombination analysis revealed a large number of potential recombination events that were scattered across the genome. As PRRSV does not contain typical recombination hot-spots as seen in other viruses, WGS-based typing is preferred over ORF-based typing, as it allows for identifying recombination events across the entire genome. Moreover, the full genetic diversity among PRRSV strains can only be captured by analysing (nearly) complete genomes. Unfortunately, the routine use of WGS as a typing tool is still hindered by its cost and turnaround time. Further developments in sequencing technologies and bioinformatics are needed to make WGS more affordable and feasible in the veterinary field. In the meantime, the accuracy of ORF-based typing can be improved by including multiple regions. Finally, we also identified several vaccine-derived recombinant strains, which once more raises the question of the safety of these vaccines.

## Figures and Tables

**Table 1:** Number of InDels found exclusively in the Belgian dataset or in the complete dataset.

ORF	Protein	Belgian Dataset Internal/Terminal InDels	Complete Dataset Internal/Terminal InDels
ORF1ab	NSP1	0/0	6/0
ORF1ab	NSP2	50/0	122/0
ORF1ab	NSP3	0/0	0/0
ORF1ab	NSP4	0/0	0/0
ORF1ab	NSP5	0/0	0/0
ORF1ab	NSP6	0/0	0/0
ORF1ab	NSP7α	0/0	0/0
ORF1ab	NSP7β	0/0	1/0
ORF1ab	NSP8	0/0	0/0
ORF1ab	NSP9	0/0	0/0
ORF1ab	NSP10	0/0	1/0
ORF1ab	NSP11	0/0	0/0
ORF1ab	NSP12	1/5	1/7
ORF2	GP2	0/1	1/1
ORF3	GP3	9/12	18/15
ORF4	GP4	11/0	22/0
ORF5	GP5	0/0	0/0
ORF6	M	1/0	1/0
ORF7	N	0/0	2/2

Internal InDels are the result of the insertion or deletion of one or more codons within an ORF, whereas terminal InDels are due to the introduction or disruption of a stop codon.

**Table 2:** Vaccine-derived recombinants found among the Belgian PRRSV-1 sequences.

Event	Recombinant	Major Parent	Minor Parent	Start/Stop Position	Region Exchanged
7	MZ417409	MZ417402	KT988004	11,927/13,412	ORF2-ORF5
15	MZ417409	MZ417402	KT988004	13,413/14,733	ORF5-ORF7
5	MZ417426	MZ417421	DD093450	6049/9047	ORF1a-ORF1b
36	MZ417449	MZ417446	KT988004	8104/8543	ORF1b
9 ^a^	MZ417459	MZ417446	DD093450	6514/8680	ORF1a-ORF1b
13	MZ417463	MZ417462	KT988004	13,157/14,587	ORF5-ORF7
2	MZ417464	MZ417463	DD093450	2721/10,884	ORF1a-ORF1b
13	MZ417464	MZ417462	KT988004	13,232/14,587	ORF5-ORF7
3	MZ417469	MZ417467	GU067771	6271/10,979	ORF1a-ORF1b
51	MZ417469	MZ417467	GU067771	1/542	ORF1a
20 ^b^	MZ417496	MZ417417	KT988004	13,366/14,695	ORF5-ORF7

Potential recombination events were identified using RDP5.05 [51]. Sequences were considered to be recombinant when the same recombination signal was detected by at least 4 methods. The start/stop positions refer to the position in the recombinant sequence of the WGS dataset, which does not contain the terminal untranslated regions (see File S2 for more details). ^a^ The same recombination event was detected in sequences MZ417456- MZ417458. ^b^ The same recombination event was detected in sequence MZ417495.

**Table 3:** Phylogenetic signal present in the complete PRRSV-1 datasets.

Dataset	Length Alignment	% Fully Resolved Quartets	% Partly Resolved Quartets	% Unresolved Quartets
CDS	15,591	97.65	2.24	0.11
ORF1a	7506	94.85	4.53	0.62
ORF1b	4458	89.26	8.81	1.93
ORF2a	747	66.06	15.89	18.05
ORF3	810	74.79	15.28	9.92
ORF4	555	66.94	19.58	13.48
ORF5	603	82.62	9.20	8.18
ORF6	519	75.04	13.67	11.29
ORF7	393	61.40	13.85	24.75

The phylogenetic content of each dataset was evaluated by likelihood mapping analysis [35] as implemented in IQ-TREE v2.1.0 [32].

**Table 4:** Comparison of the renumbered clustering schemes of the CDS and ORF datasets.

Dataset	AMI	F(b^3^)
CDS/ORF1a	0.93	0.90
CDS/ORF1b	0.90	0.86
CDS/ORF2a	0.81	0.75
CDS/ORF3	0.80	0.71
CDS/ORF4	0.74	0.68
CDS/ORF5	0.79	0.73
CDS/ORF6	0.74	0.65
CDS/ORF7	0.73	0.65

Clustering schemes of the ORF datasets were compared with the CDS clustering scheme, which served as reference. The adjusted mutual information (AMI) and BCubed F-score (F(b^3^)) were calculated using the R packages aricode [49] and DPBBM [50], respectively.

## Data Availability

All genome sequences have been deposited in GenBank under accession numbers MZ417390 to MZ417500.

## Supplementary Materials

The following are available online at https://www.mdpi.com/article/10.3390/v13122419/s1, Table S1: Metadata of the samples that were sequenced in this study, Table S2: Maximum and mean pairwise genetic distances of the Belgian (excluding vaccine-like strains), non-Belgian (subtype I and subtypes I–III strains) and complete (all strains) PRRSV-1 datasets, Table S3: Clustering schemes and cluster codes for each PRRSV strain, File S1: Gap patterns and gap codes for each PRRSV strain, File S2: Recombination events in the WGS dataset.
